# Direct Competitive Kinetic Isotope Effect Measurement Using Quantitative Whole Molecule Matrix‐Assisted Laser Desorption Ionization Time‐of‐Flight Mass Spectrometry

**DOI:** 10.1002/cbic.202500539

**Published:** 2026-06-24

**Authors:** Teodora Kljaic, Merritt A. Scott, Veronica Guirguis, Michal Tyrlik, Andrew Liu, Myles B. Poulin

**Affiliations:** ^1^ Department of Chemistry & Biochemistry University of Maryland at College Park College Park Maryland USA

**Keywords:** *β*‐galactosidase, enzymology, kinetic isotope effect, matrix‐assisted laser desorption ionization time‐of‐flight, mass spectrometry

## Abstract

Kinetic isotope effect (KIE) measurements are a powerful tool to interrogate the microscopic steps in enzyme catalyzed reactions and can provide detailed information about transition state structures. However, the application of KIE measurements to study enzymatic reactions is not widely applied due to the tedious and complex analytical workflows required to measure KIEs with sufficient precession. Here, we report a method for the direct measurement of competitive KIEs using a whole molecule matrix‐assisted laser desorption ionization (MALDI) time‐of‐flight (TOF) mass spectrometry (MS). Using isotope labeled internal standard introduced when quenching the enzyme reaction at multiple time points enables the simultaneous measurement of both the relative heavy/light isotope ratio *R* and fractional conversion *F* relative to the internal standard for each sample as the reaction progresses. We applied this approach to measure both [1′‐^13^C]lactose and [6′‐^13^C]lactose KIEs for the *E. coli* *β*‐galactosidase (LacZ) catalyzed hydrolysis of lactose. This MALDI‐TOF MS based KIE approach can measure enzymatic KIEs with precision comparable to those obtained using competitive radioisotope labeling and NMR‐based approaches.

## Graphical abstract

Kinetic Isotope effect (KIE) measurements are a powerful tool to interrogate enzyme mechanisms and transition state structures. In this study, we develop a simple quantitative MALDI‐TOF mass spectrometry approach to measure competitive heavy atom KIEs. This approach continuously measures the change in isotope ratio (*R*/*R*_‖_) as a function of reaction fractional conversion (*F*) to obtain highly reproducible KIE measurements.

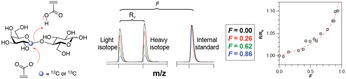

## 1 Introduction

The measurement of kinetic isotope effect (KIEs) is among the most powerful tools available for the study of enzyme reaction mechanisms [1, 4]. Highly precise KIE measurements can provide unique insight into the rate limiting microscopic steps of enzyme catalyzed reactions [1, 5] and can facilitate detailed atomistic characterization of transition state structures to guide the design of incredibly potent transition state analog enzyme inhibitors [6, 9]. Despite the utility of KIE measurements for examining enzyme mechanisms, they have not been widely used by the broader enzymology research community. This is due, at least in part, to the complex analytical workflows required to measure heavy atom KIEs using available approaches and difficulty obtaining suitably labeled substrates for these KIE measurements. While primary hydrogen atom and solvent KIEs are more common, few labs conduct the precise heavy atom KIEs required for detailed analysis of enzyme transition state structures.

KIEs for heavy atoms (i.e., C, N, O, S, etc.) are of low magnitude with typical values falling in the range of 0.95–1.05, necessitating highly precise analytical measurements [3, 10, 11]. Typically, these are measured under competitive conditions giving isotope effects on the enzyme specificity constant *V*/*K* that report on all isotopically sensitive steps up to and including the first irreversible step of the reaction.

In competitive KIE measurements, two isotopologues of the same substrate—one “light” and one isotopically labeled “heavy”—react in the same reaction mixture. As the reaction proceeds, the isotopologue that reacts faster becomes depleted relative to the slower reacting isotopologue. Monitoring the change in isotope ratios of the remaining substrate over the course of the reaction therefore allows determination of which isotopologue is preferentially consumed and provides the kinetic isotope effect associated with the isotopically sensitive step. Such competitive KIEs can be calculated using Equation (1):

(1) $$\left(\frac{R_{F}}{R_{o}}\right)={\left(1-F\right)}^{\left(\frac{1}{\mathrm{kie}}-1\right)}$$

where *R*_F_ is the heavy/light isotope ratio measured for the unreacted substrate at *F*, *R*_o_ is the initial heavy/light isotope ratio for the substrate at time zero, *F* is the fraction of the light isotopologue substrate that has been converted to product (i.e., fractional conversion), and kie is the *V*/*K* isotope effect. Thus, the determination of competitive KIEs requires precise measurement of both the analyte concentration (to determine *F*) and heavy/light isotope ratios (to determine *R*_F_ and *R*_o_).

The most common approaches to measure heavy atom KIEs utilize direct competitive isotope ratio measurements using a pair of isotope labeled substrates by liquid‐scintillation counting [12, 19], isotope ratio mass spectrometry [20, 24], or more recently nuclear magnetic resonance (NMR) spectroscopy [25, 29]. While these approaches can be successfully employed to measure heavy atom KIEs for enzymatic reactions, they require complex analytical workflows to purify and isolate the analyte prior to analysis, or in the case of competitive NMR KIE measurements, they require high amounts of multiple isotopically labeled reactants.

As an alternative, direct whole molecule mass spectrometry (MS) has emerged as a promising approach for heavy atom KIE measurements due to its high sensitivity and ability to distinguish multiple isotopes simultaneously [3, 30, 38]. Generally, isotopologues of an analyte ionize with the same ionization efficiency allowing for relative quantification of their isotope ratios. Another advantage of whole molecule MS measurements is that they enable the analysis of stable isotope labeled analytes that are generally more easily available and more affordable than radioisotope labeled materials required for scintillation counting. However, the technique is not without its limitations. First, MS‐based KIE measurements have traditionally examined enzyme reaction at partial conversion to quantify isotope ratio *R*_F_ for the reactant or product that have already been purified from the reaction mixture [30, 32, 33, 35, 36, 38, 39], necessitating complex analytical workflows for the isolation and purification of the analyte prior to analysis. Second, slight variations in sample composition can affect ionization efficiency of an analyte making the precise and accurate quantification of analyte concentrations required for determining fractional conversion *F* challenging. As a result, an alternative approach like UV‐Vis spectroscopy, or high‐performance liquid chromatography (HPLC) will frequently be carried out in parallel to quantify the analyte concentration for the calculation of *F*. These parallel approaches provide only an approximate measurement of *F* as they do not distinguish between the conversion of the heavy and light isotopologues. Thus, we sought to simplify the procedure to enable both *R*_F_ and *F* to be determined quantitatively in a single measurement.

**FIGURE 1:**
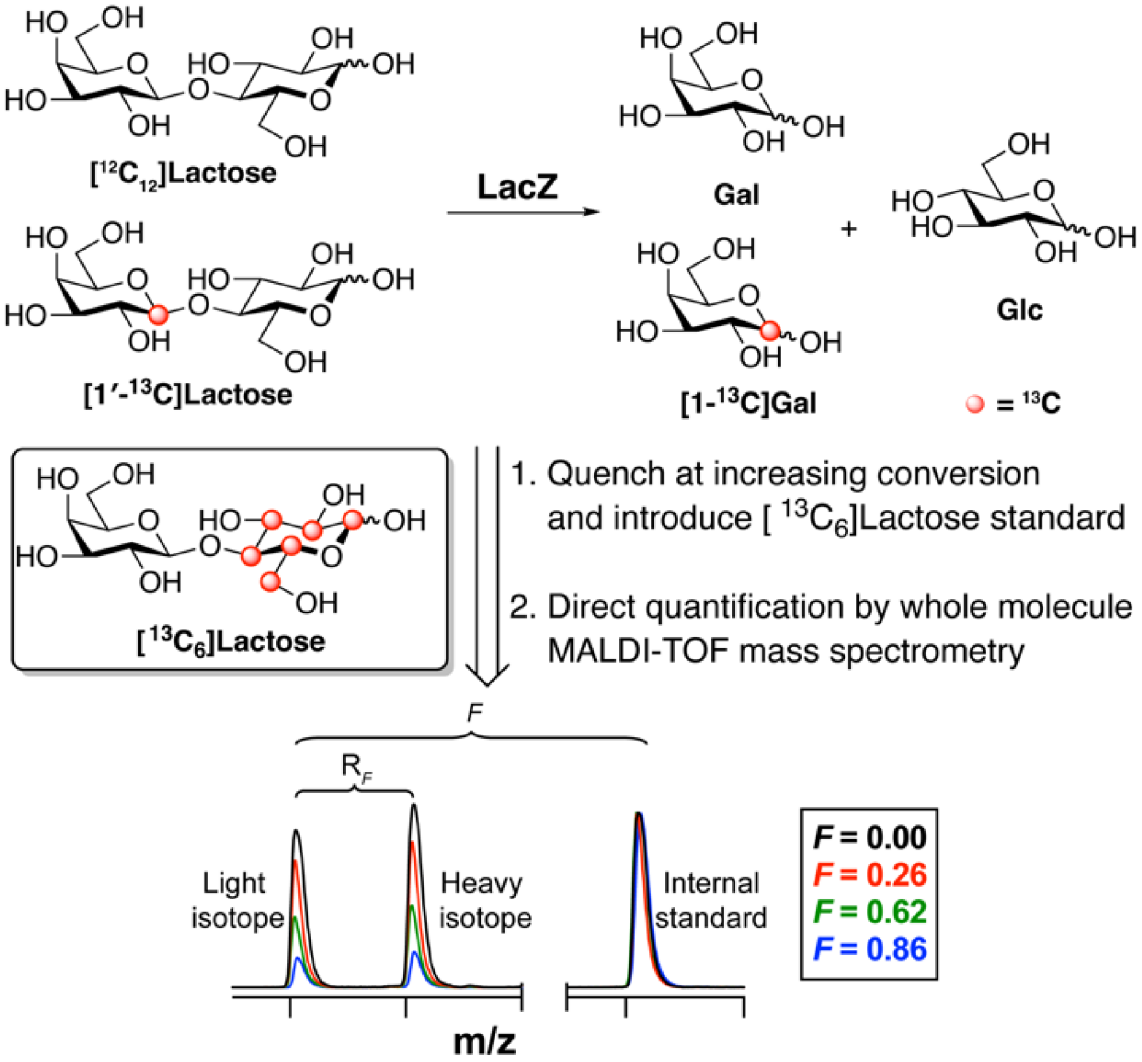
Quantitative MALDI‐TOF MS approach for competitive KIE measurements. An enzymatic reaction containing light ([^12^C_12_]lactose) and heavy ([1′‐^13^C]lactose) is allowed to proceed and aliquots are quenched at increasing fractional conversion. An internal standard ([^13^C_6_]lactose) is added during the quench. Isotope ratios (*R*_F_) and fractional conversion (*F*) are determined at each time point from the heavy/light isotope peak area and the light/internal standard peak area, respectively.

Here, we report a method that enables the direct measurement of competitive KIEs using a quantitative whole molecule matrix‐assisted laser desorption ionization (MALDI) time‐of‐flight (TOF) MS approach that does not require isolation and purification of the reactants prior to analysis (Figure 1). This approach enables quantitative measurements of both relative isotope abundance of an analyte and fractional conversion of the light isotopologue (*F*) in single measurements. This is accomplished by introducing a known concentration of internal standard, which has the same structure as the analyte but a different isotope labeling pattern, to quenched samples of the enzyme reaction. By quenching the enzyme reaction at multiple time points, it enables us to measure *R*_F_ and *F* relative to the internal standard for each sample as the reaction progresses. These values can then be fit using Equation (1) to obtain highly precise *V*/*K* KIEs. MALDI was selected as the ionization source for these measurements as it is more tolerant of salts and buffer composition than electrospray ionization (ESI) enabling the direct analysis of enzyme reaction mixtures without requiring isolation or purification of the analyte prior to the analysis. While MALDI‐TOF MS has been previously employed for KIE measurements [39, 40], what distinguishes our approach is the inclusion of an internal standard during sample quenching, which enables the simultaneous determination of *R*_F_ and *F* in a single measurement.

## 2 Results and Discussion

To evaluate the utility of this MALDI‐TOF MS approach for enzymatic KIE measurements, we chose to examine the hydrolysis of lactose catalyzed by *E. coli* *β*‐galactosidase (LacZ) as a model system. The structure and chemical mechanism of LacZ have been extensively studied [41, 46], and ^18^O, secondary‐^2^H, and solvent KIE measurements have been previously reported [33, 41, 47]. LacZ is a retaining glycosyl hydrolase that catalyzes the hydrolysis of lactose with an overall retention of the anomeric configuration. Despite the substantial efforts that have been made to elucidate the mechanism of LacZ, there have been no previous KIE studies that have used the native lactose as the substrate. Here, we used our quantitative MALDI‐TOF MS approach to measure both 1′‐^13^C and 6′‐^13^C KIEs for the LacZ catalyzed hydrolysis of lactose.

**FIGURE 2:**
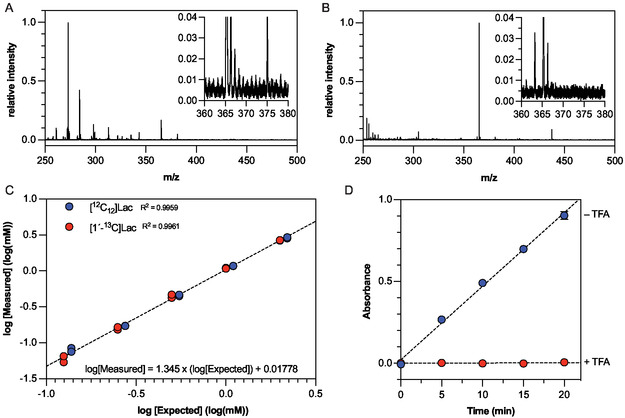
Optimization of MALDI‐TOF MS conditions for competitive KIE measurements. (A) A sample of [^12^C_12_]‐lactose analyzed using DHB as the matrix. (B) The same sample of [^12^C_12_]‐lactose analyzed using graphene as the matrix. The spectra were normalized to the intensity of the largest ion peak within the *m*/*z* window analyzed. (C) Standard curves for [^12^C_12_]‐lactose and [1′‐^13^C_1_]‐lactose concentrations measured relative to a fixed 500 μM concentration of [^13^C_6_]‐lactose internal standard. (D) Measurement of LacZ quenching efficiency. Samples of PNP‐Gal and LacZ were either pre‐quenched with TFA or quenched at the times indicated. The absorbance of the pre‐quenched samples was compared to the unquenched at each time point.

The experiments were initiated using *β*‐lactose. Under aqueous conditions lactose undergoes mutarotation between *α* and *β* anomers. However, this interconversion occurs rapidly relative to the timescale of the enzymatic reaction, resulting in an equilibrated mixture of anomers during the course of the experiment. Our initial application of this MALDI‐TOF MS approach to measure LacZ KIEs made use of 2,4‐dihydroxybenzoic acid (DHB) as a matrix compound. However, the use of DHB suffered from matrix ion interference and detector saturation, limiting the accurate quantification of lactose concentration (Figure 2A). Specifically, we observed periodic baseline fluctuations within the *m*/*z* window of interest for the ionization of lactose (*m/* *z* 360–380), that overlapped with our analyte peaks. The intensity of these baseline fluctuations varied between samples making accurate quantification of the analyte peak area impossible. To overcome these limitations, we switched to the use of graphene as a macromolecular matrix compound (Figure 2B). This effectively removed interference due to matrix ions and resulted in a more regular baseline. Moreover, the lactose–Na^+^ adduct was observed as the major peak within our mass spectra using this graphene matrix. Samples of lactose prepared in 20 mM Tris‐HCl buffer (pH 7.4) with 100 mM NaCl demonstrated optimum ionization of a lactose–Na^+^ adduct without signal suppression and provides a stable pH for efficient LacZ enzyme activity. We found that averaging the peak areas from five technical replicates of the same sample provided better precision of the isotope ratio measurements and were sufficient to obtain accurate estimates of the standard deviation in the measurements (Figure S1). Increasing the number of technical replicates beyond five did not significantly impact the standard deviation of the measurements, and so the average peak areas from five technical replicates were used to calculate analyte concentrations in all subsequent experiments.

To ensure precise and accurate analyte concentrations could be measured using this approach, we prepared standard curves for both [^12^C_12_]lactose and [1′‐^13^C]lactose measured relative to a fixed concentration of [Glc‐^13^C_6_]lactose. Interestingly, we observed a non‐linear relationship between the peak area and analyte concentration that was consistent for both [^12^C_12_]lactose and [1′‐^13^C]lactose. This nonlinearity in dynamic range is well known for MALDI‐TOF measurements and are historically fit using a power law relationship in the form *y* = *k*(*x*)*^n^* [48]. By plotting the log(peak area) versus log(analyte concentration), we can display this power law relationship as a linear graph in the form log(*y*) = *n* log(*x*) + *k*. Importantly, we found that the data for both isotopologues [^12^C_12_]lactose and [1′‐^13^C]lactose followed the same power law relationship, and a linear fit of the log–log plot allows us to accurately estimate analyte concentrations over a more broad dynamic range (Figure 2C and S2), and this standard curve was used to calculate analyte concentrations based on peak area in all subsequent KIE experiments.

**FIGURE 3:**
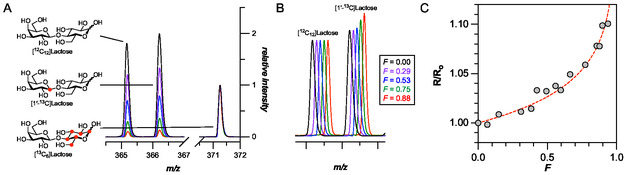
Quantitative MALDI‐TOF MS approach for competitive KIE determination. (A) Representative mass spectra normalized to the peak intensity of [^13^C_6_]lactose internal standard for the measurement of a LacZ [1′‐^13^C] KIE measured at different *F*. The peaks for [^12^C_12_]lactose, [1′‐^13^C]lactose, and [^13^C_6_]lactose are indicated. (B) The same mass spectra normalized relative to the [^12^C_12_]lactose peak intensity. (C) Representative plot of *R*/*R*_o_ vs. *F* fit to Equation (1) using 15 time points derived from a single LacZ reaction. Additional curve fitting for [1′‐^13^C]lactose and [6′‐^13^C]lactose KIEs are shown in supporting information Figure S3.

Using the optimized MALDI‐TOF MS conditions, LacZ KIEs were measured using [1′‐^13^C]lactose and [6′‐^13^C]lactose. For each KIE measurement, a minimum of fifteen time points were analyzed from each individual reaction mixture at varying fractional conversion *F*, where the enzymatic activity of LacZ was quenched through the addition of 3 mM trifluoroacetic acid (TFA). At the same time 500 μM of [^13^C_6_]lactose internal standard was introduced. These conditions were found to effectively inhibit the LacZ activity, as seen in Figure 2D, without interfering with subsequent MALDI‐TOF MS analysis. Figure 3A shows representative mass spectra measured at *F* ranging from 0.0 to 0.88 that have been normalized to the peak intensity of the [^13^C_6_]lactose standard. The peaks for unlabeled [^12^C_12_]lactose (*m/z* 365.1) and [1′‐^13^C]lactose (*m*/*z* 366.1) decrease as a function of increasing *F* relative to that of the [^13^C_6_] lactose internal standard (*m/z* 371.1). When these same spectra are instead normalized to the intensity of the [^12^C_12_] lactose peak, a clear increase in the [1′‐^13^C]lactose relative to the [^12^C_12_]lactose is observed as a function of increasing *F* (Figure 3B) consistent with a normal KIE value. The data from all 15 time points were fit using Equation (1) as shown in Figure 3C to calculate individual *V*/*K* KIE values. Four individual KIE measurements were carried out on separate days, and the individual KIEs determined by independently fitting the respective *R*/*R*_o_ versus *F* data from each experiment to Equation (1) are summarized in Table 1 and Figure S3, resulting in an average primary [1′‐^13^C] KIE of 1.034  ±  0.005. The precision of these KIE measurements is comparable to those obtained using competitive radioisotope labeling and NMR‐based approaches [12, 26, 27].

**TABLE 1:** Summary of LacZ KIEs.

KIE	Experimental KIE^a^	Average^b^
1′‐^13^C	1.034 ± 0.005	1.034 ± 0.005
	1.028 ± 0.002	
	1.039 ± 0.002	
	1.036 ± 0.002	
6′‐^13^C	0.999 ± 0.007	1.001 ± 0.002
	1.001 ± 0.003	
	1.003 ± 0.005	
	1.002 ± 0.009	

a Error is the standard error for the nonlinear regression fit.

b Mean and standard deviation for four KIE experiments.

To verify the primary [1′‐^13^C] KIE is not an artifact of the MALDI‐TOF MS analysis method, we additionally measured a LacZ KIE using [6′‐^13^C] lactose. The 6′‐carbon of lactose is positioned distal from the reaction center and not isotopically sensitive to the reaction catalyzed by LacZ. As summarized in Table 1, we observed an average [6′‐^13^C] lactose KIE of 1.001 ± 0.002, which is not significantly different from the expected value of 1.

**FIGURE 4:**
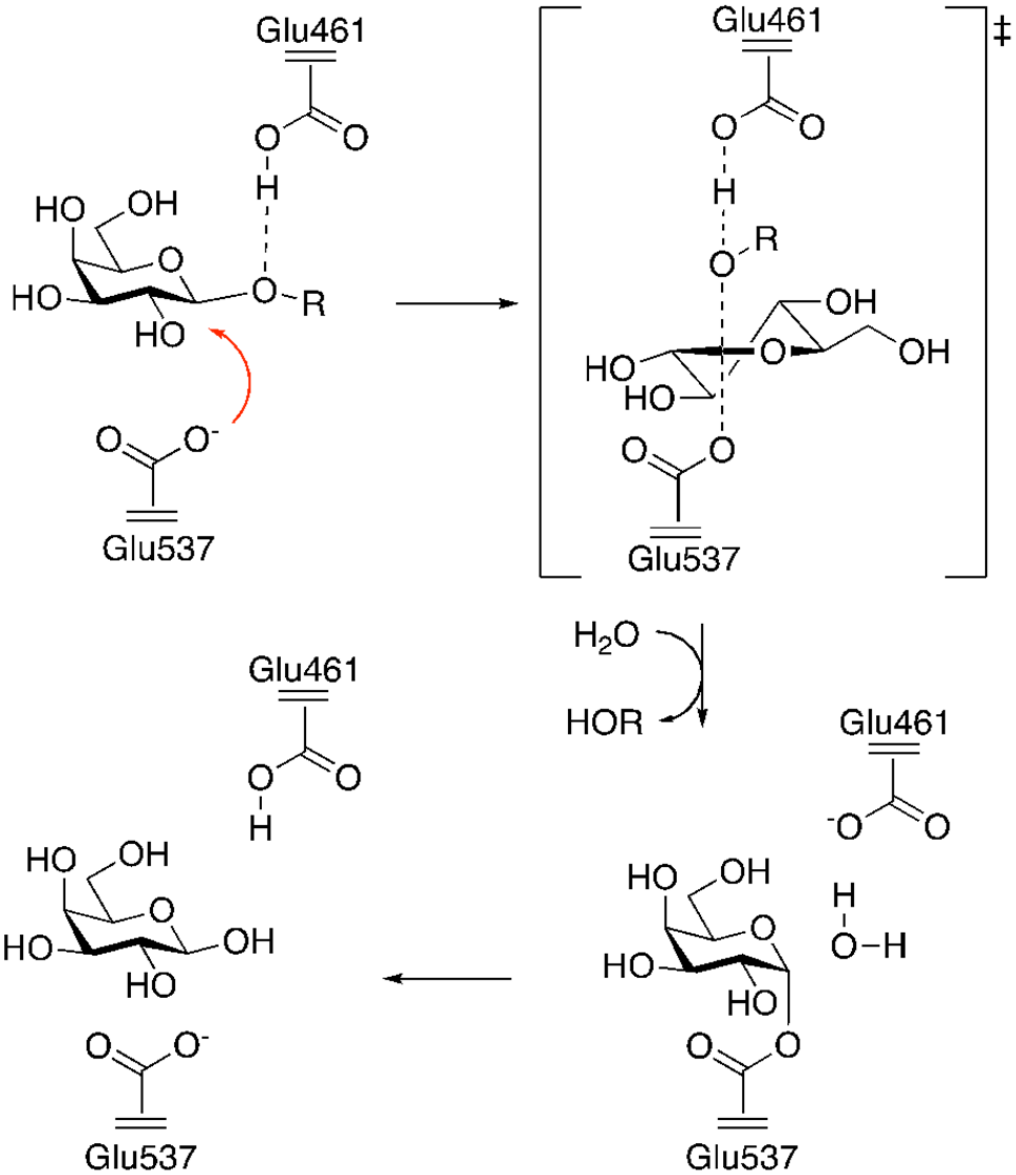
Mechanism for LacZ catalyzed hydrolysis of lactose highlighting the first TS.

Both the [1′‐^13^C]lactose and [6′‐^13^C]lactose KIE are consistent with the accepted mechanism for LacZ, which involves an initial S_*N*_2 attack of Glu537 to form a covalent glycosyl–enzyme intermediate and release glucose, followed by hydrolysis of the glycosyl–enzyme intermediate via a nucleophilic attack by water (Figure 4) [44]. Previous leaving group ^18^O and secondary ^2^H KIE measurements measured for LacZ using phenyl‐glycoside analogs show that the initial attack of Glu537 represents the first ‘irreversible’ step in the reaction [33, 41]. The magnitude of the [1′‐^13^C]lactose KIE measured here is also consistent with an attack of Glu537 being effectively irreversible under these experimental conditions. For retaining glycosidases, primary ^13^C kinetic isotope effects at the anomeric carbon are typically expected to fall in the range of ~1.02–1.05 when significant bond cleavage to the leaving group occurs in the transition state. The experimentally measured value of 1.034 therefore falls within the range predicted for an S_*N*_2‐type nucleophilic attack at the anomeric carbon leading to formation of a covalent glycosyl–enzyme intermediate.

## 3 Conclusion

The results presented herein demonstrate the utility of this MALDI‐TOF MS based KIE measurement approach. This approach can easily be adapted for the analysis of different analytes and enables simultaneous measurement of heavy/light isotope ratio *R*_F_ and fractional conversion *F* in a single measurement without requiring isolation and purification of the analyte prior to analysis. This enables the use of easily accessible stable isotope labeled samples, while obtaining KIEs with comparable precession to competitive radioisotope labeling methods, yet it is not without its limitations. For instance, the accurate determination of fractional conversion requires the addition of an isotopically labeled internal standard with a distinct *m*/*z* from the isotopologues being kinetically analyzed. Uniformly labeled standards are available for many common metabolites, but such standards may not be readily accessible for all analytes of interest. In this study, we used a [^13^C_6_]lactose isotopologue as the internal standard but any isotopologue with a unique mass could be employed for this approach. A second limitation is that MALDI‐TOF analysis of low molecular weight compounds can be complicated by matrix ion interference, particularly below *m*/*z* ~300. Although this was not problematic for lactose using graphene as a matrix, alternative ionization strategies or matrices may be required for smaller analytes. It is also important to note that while we used MALDI as an ionization source to enable the direct analysis of enzyme reaction mixtures without requiring isolation and purification of the analyte, in principle this quantitative whole molecule MS approach can be applied using any ionization method.

## 4 Experimental Section

### 4.1 General

Unless otherwise noted, all chemicals were purchased as analytical or reagent grade and used without further purification. Isotopically labeled [^13^C_6_] lactose, [1′‐^13^C] lactose, and [6′‐^13^C] lactose were purchased from Omicron Biochemicals, Inc and prepared as described below. MALDI‐TOF mass spectrometry measurements were recorded on a Bruker Autoflex Speed spectrometer equipped with a 2 KHz smartbeam II laser, with a time of flight (TOF)‐analyzer capable of both positive and negative ion mode as described in detail below. Matrix solutions of 2,5‐dihydroxybenzoic acid (DHB) were prepared in a 50:50 mixture of acetonitrile:1% aqueous trifluoroacetic acid (TFA) at a final concentration of 10 mg/mL. Graphene matrix samples were prepared as described previously [49]. Briefly, graphite oxide was prepared following the Hummers method [50], starting from commercial graphite powder. Graphene was then prepared via the reduction of graphite oxide with hydrazine [51]. The reduced graphene was obtained as a black precipitate that were prepared fresh for MALDI‐TOF MS by suspending in 100% ethanol to a final concentration of 0.1 mg/mL [49].

### 4.2 Protein and Substrate Preparation

Commercial *β*‐galactosidase (LacZ) from *Escherichia coli* was obtained as a lyophilized powder and prepared in 20 mM sodium phosphate buffer pH 7.1 to a final concentration of 1 mU/µL, where 1 U is defined as the amount of enzyme required to hydrolyze 1.0 μM of 2‐nitrophenol *β*‐D‐galactopyranoside per minute at pH 7.1 and 37°C. Individual aliquots of enzyme were flash frozen and stored at −80°C until use.

Lactose substrate samples, both isotopically labeled and unlabeled, were prepared by dissolving the solid to a concentration of 10 mM.

### 4.3 Sample Quenching

Quenching conditions that enable the rapid inactivation of enzyme activity were tested using a colorimetric LacZ activity assay using 4‐nitrophenol *β*‐D‐galactopyranoside (PNP‐Gal) as a substrate, by measuring the absorbance of the PNP product at 410 nm. First, a 500 μL reaction mixture was prepared containing 1 mM PNP‐Galactose, 1 mM MgCl_2_ in 20 mM Tris‐HCl buffer pH 7.1. A second 500 μL reaction mixture containing 1 mM PNP‐Galactose, 1 mM MgCl_2_, 20 mM Tris‐HCl buffer pH 7.1, was set up in parallel and pre‐quenched with 6 mM TFA. A 100 μL fraction from each reaction mixture was removed prior to initiating the reaction to allow for measurement of background absorbance at time zero. Reactions were initiated through the addition of 833 μU of *β*‐galactosidase to each reaction mixture and allowed to proceed at 22°C. At 5 min time intervals, 100 μL aliquots were removed from each reaction mixture and diluted with an equal volume of quench solution. For the first reaction mixture, the quench solution consisted of 6 mM aqueous TFA, whereas the quench solution for the second reaction mixture contained only water. The appropriate quench solution was also added to the time zero time points. Immediately before measuring the absorbance at 410 nm, the pH of all of the samples were adjusted by adding 100 μL of 1 M NaOH, and samples were transferred to a clear bottom 96 well microtiter plate. End point absorbance measurements were recorder on a Spectramax M5 multimode plate reader (Molecular Devices, USA), and reaction was repeated in triplicate.

### 4.4 MALDI‐TOF MS Sample Preparation and Acquisition

For MALDI‐TOF measurements using DHB as the matrix, samples were prepared by spotting 1 µL DHB per spot on the target plate followed by drying for 10 min at room temperature. Next, 1 µL of analyte, prepared as outlined below, was spotted over the dry matrix and allowed to dry for an additional 10 min at room temperature and humidity of 45%–50%.

For MALDI‐TOF measurements using graphene as the matrix, 10 µL of analyte sample was directly mixed with 10 µL of graphene suspension and subsequently dispersed for 10 min in a sonication bath. The 1 µL samples of the graphene–analyte mixture were spotted on the target plate and allowed to dry for 10 min at room temperature and humidity of 45%–50%.

Mass spectra were recorded in positive‐ion reflectron mode to achieve optimal baseline peak resolution. A spectral window from 280 to 580 *m*/*z* was used for analyzing lactose samples. Sodium chloride (25 mM) was included in the analyte sample buffer to boost the sensitivity for detection of lactose + Na^+^ molecular ions. A total of five technical replicate spots were analyzed for each analyte sample to minimize variability in sample concentration or isotope composition resulting from sample spotting. Summed spectrum from a minimum of 3000 shots for each sample spot were recorded and exported in mzXML format and further processed in *R* using the MaldiQuant package [52], as described below.

### 4.5 Standard Curve for Quantification of Analyte Concentration Versus Peak Area by MALDI‐TOF MS

To calibrate the relationship between analyte concentration and MALDI‐TOF MS peak area, standards were prepared by mixing varying concentrations of lactose or [1′‐13C]lactose with a fixed concentration (500 µM) of [13C6]lactose internal standard. Lactose or [1′‐^13^C]lactose were analyzed at concentrations of 2, 1 mM, 500, 250, and 125 μM in 20 mM Tris‐HCl pH 7.1 containing 10 mM MgCl_2_ and 100 mM NaCl. Each standard was then mixed in a 1:1 ratio with either a 1 or 0.5 mM solution of [^13^C_6_]lactose standard in 6 mM aqueous TFA. Two internal standard concentrations were used to ensure that calculations of analyte concentration were consistent when using different concentrations of internal standard. Each sample was then prepared for MALDI‐TOF measurements using graphene as the matrix as described above. A total of five separate spots for each standard concentration were prepared for the measurements. Summed spectrum from a minimum of 3000 shots for each sample spot were recorded and exported in mzXML format and further processed in *R* using the MaldiQuant package [51], as described below. The observed concentration for each stock solution was then calculated using the ratio of the peak area for the analyte sample divided by the peak area of the [^13^C_6_]lactose standard and multiplied by the concentration of the [^13^C_6_]lactose standard. The plots of [observed] vs [expected] for both [^12^C_12_]lactose and [1′‐^13^C]lactose were found to be nonlinear over the concentration range tested but gave the same values independent of the concentration [^13^C_6_]lactose standard used.

### 4.6 KIE Measurements

Reaction mixtures for LacZ KIE measurements were prepared containing 2 mM total lactose substrate (in a ~1:1 ratio of “heavy” to “light” sample) in 20 mM Tris‐HCl pH 7.1 with 10 mM MgCl_2_ and 100 mM NaCl in a final volume of 120 μL. Prior to initiation of the reaction, a time zero time point consisting of 20 μL of the reaction mixture was removed and diluted to a final volume of 22 μL to measure initial lactose concentrations and isotope ratios (*R*_o_). Reactions were carried out at 30°C and initiated through the addition of 10 μL of 166 mU/mL *β*‐galactosidase. At regular time intervals, 5 μL aliquots of the reaction mixture were removed, and the LacZ activity was quenched by mixing with an equal volume of quench solution consisting of 0.5 mM [Glc‐^13^C_6_]lactose standard in 6 mM aqueous TFA. These conditions were found to rapidly inactivate *β*‐galactosidase without any degradation of the lactose substrate. Quenched fractions were centrifuged at 17,000 g for 2 min to remove any solid precipitates and stored at −20°C prior to MALDI‐TOF analysis as described above. A reaction mixture containing only “light” lactose was analyzed in the same fashion to account for concentration of natural abundance ^13^C present in the “light” lactose sample. The peak area for the [M + 1]^+^ peak (366 *m*/*z*) was used to determine the natural abundance ^13^C present in the “light” lactose sample. There are 12 carbons in lactose that are assumed to contribute equally to the observed concentration of natural abundance ^13^C. To account for these naturally abundant ^13^C within our KIE measurements.

### 4.7 Peak Integration and Data Analysis

Relative peak areas for the sodium adducts of [^12^C_12_]Lac (light, *m*/*z* = 365.2), [^13^C_1_]Lac (heavy, *m*/*z* = 366.2) and [^13^C_6_]Lac (standard, 371.2) were determined for each mass spectrum using a numerical peak integration script developed in *R*. Briefly, mass spectral mzXML files were imported in *R* using the MALDIquant package [52]. A 10 *m*/*z* window containing the peaks of interest was selected for further analysis. The 25^th^ percentile of all intensity values within this segment was used as a baseline and subtracted from all intensities within the segment. Subsequently, the boundaries of each relevant peak were identified. Initial guesses of outer integration boundaries were provided by the user and were identical for all mass spectra analyzed from the same enzyme‐substrate combination. Subsequently, an approximate first derivative was calculated for the entire spectrum segment and smoothed using an “SMA” moving average calculation. A difference in treatment of the left and right boundaries was necessitated by the peak asymmetry. The left boundary was selected as the highest mass number lower than the 20^th^ percentile of smoothed derivatives within the segment bound by the initial guess and the peak maximum. The right boundary was selected as the lowest mass number lower than the 5^th^ percentile of smoothed derivatives within the segment bound by the peak maximum and the initial guess. The total peak intensity was determined after boundary selection from the sum of all intensities within these boundaries. A plot of spectra with overlaid final integration boundaries was manually examined for each measurement to verify accurate boundary detection. An integration was considered successful if the boundaries included the entire target peak, excluded all other peaks, and divided overlapping peaks at a local minimum between them. Only spectra in which the peaks for [^12^C_12_]lactose (light, *m*/*z* = 365.2), [^13^C_1_]lactose (heavy, *m*/*z* = 366.2) were fully resolved to baseline resolution were included in the analysis of KIEs.

Natural abundance ^13^C contributions to the M + 1 peak were quantified using control samples containing only unlabeled lactose. The measured M + 1/M ratio from this control spectrum was used to correct the observed heavy isotopologue signal in experimental spectra. Specifically, the expected natural abundance contribution to the *m*/*z* 366 peak arising from the unlabeled lactose isotopic distribution was subtracted prior to calculation of heavy/light isotope ratios.

## Funding

This study was supported by the National Science Foundation (CHE1945162).

## Conflicts of Interest

The authors declare no conflicts of interest.

## Supporting information

Supplementary Material

## Data Availability

The data that support the findings of this study are available from the corresponding author upon reasonable request.
