# Supplementary material for: Direct Competitive Kinetic Isotope Effect Measurement Using Quantitative Whole Molecule Matrix‐Assisted Laser Desorption Ionization Time‐of‐Flight Mass Spectrometry
Source: Chembiochem. 2026 Jun 24;27(12):e202500539. doi: 10.1002/cbic.202500539 (PMC13292192; doi:10.1002/cbic.202500539)
Supplement: Supplementary file 1 — Supplementary Material [file CBIC-27-e202500539-s001.pdf]

Supporting Information:

Direct Competitive Kinetic Isotope Effect Measurement Using Quantitative Whole Molecule MALDI-TOF Mass Spectrometry

Teodora Kljaic, Merritt A. Scott, Veronica Guirguis, Michal Tyrlik, Andrew Liu and Myles B. Poulin*

Department of Chemistry and Biochemistry, University of Maryland College Park, College Park, MD 20742, United States

*Corresponding Author: Myles B. Poulin
E-mail: mpoulin@umd.edu

Supporting Information

1. Supporting Information Figures S1 2
2. Supporting Information Figures S2 3
3. Supporting Information Figures S3 4

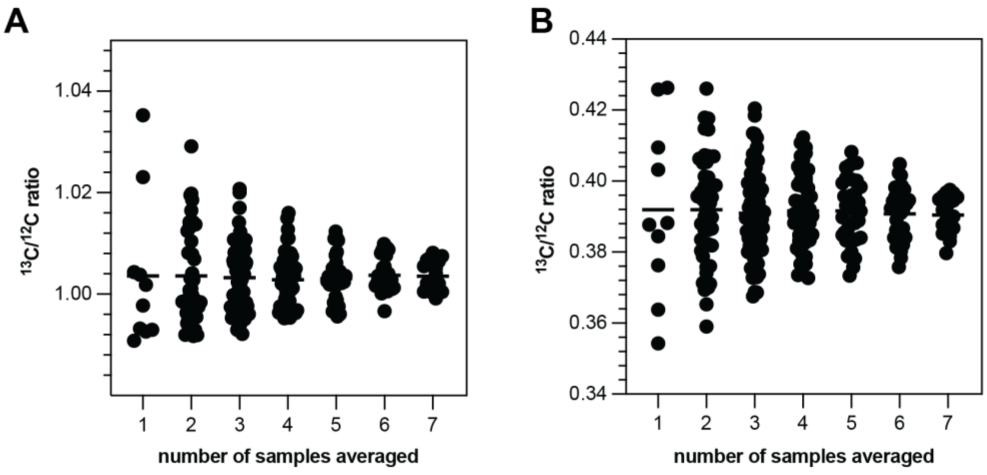

Figure S1. Determining the number of technical replicates to average to improve precision of isotope ratio measurements. $^{13}C/^{12}C$ isotope ratios were determined by averaging between 1 to 7 individual technical replicates for each data point and the precision of the measurements were analyzed by looking at the overall deviation in the determined $^{13}C/^{12}C$ isotope ratios measured at ~1:1 (A) and ~2:5 ratios of “heavy” to “light” lactose isotopologue standards (B).

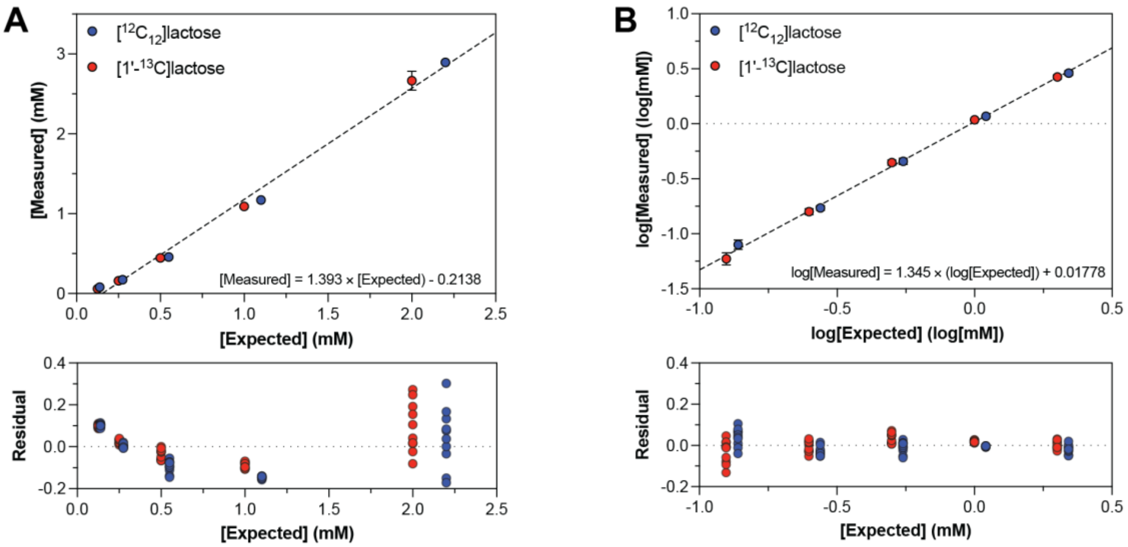

Figure S2. Standard curves for measurement of [$^{12}C_{12}$]lactose and [1′-^{13}C]lactose concentration from MALDI-TOF measurements. (A) Linear fit of [Measured] vs. [Expected] and residuals. The data shows a non-linear response. (B) Log–Log plot of Log[Measured] vs. log[Expected] and residuals. The Log–Log plot represents a power law relationship as linear.

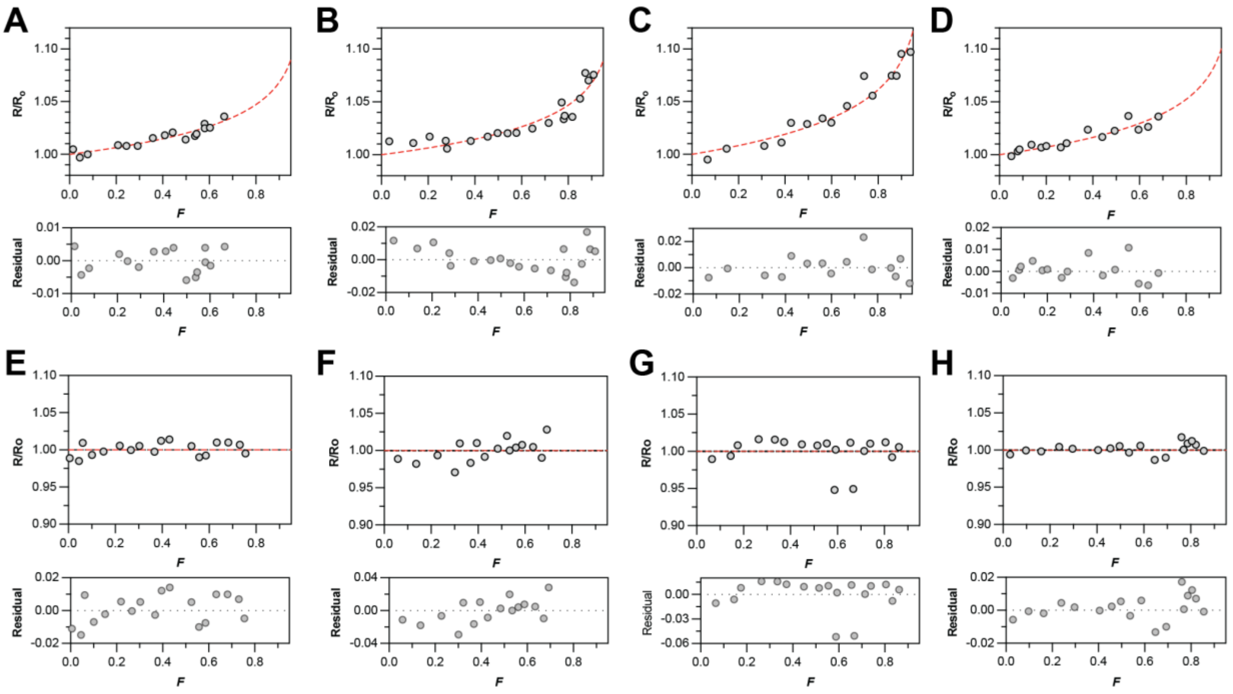

Figure S3. Individual plots of R/R_o vs. *F* fit to eq. 1 used to calculate average [1′-^{13}C]lactose (A, B, C, D) and [6′-^{13}C]lactose (E, F, G, H) KIEs that are summarized in **Table 1**. Residuals for the fit to eq. 1. are shown below each plot.
